# Reconstruction With 3D-Printed Prostheses After Sacroiliac Joint Tumor Resection: A Retrospective Case-Control Study

**DOI:** 10.3389/fonc.2021.764938

**Published:** 2022-01-04

**Authors:** Feifei Pu, Jianxiang Liu, Deyao Shi, Xin Huang, Jingtao Zhang, Baichuan Wang, Qiang Wu, Zhicai Zhang, Zengwu Shao

**Affiliations:** ^1^ Department of Orthopedics, Union Hospital, Tongji Medical College, Huazhong University of Science and Technology, Wuhan, China; ^2^ Department of Radiology, Union Hospital, Tongji Medical College, Huazhong University of Science and Technology, Wuhan, China

**Keywords:** sacroiliac joint, bone tumor, 3D printed prosthesis, lumbar iliac crest fixation, bone cement

## Abstract

**Background:**

Sacroiliac joint tumor is rare, and the reconstruction after tumor resection is difficult. We aimed to analyze and compare the clinical effects of three-dimensional (3D) printed prostheses and bone cement combined with screws for bone defect reconstruction after sacroiliac joint tumor resection.

**Methods:**

Twelve patients with sacroiliac joint tumors who underwent tumor resection and received 3D-printed prostheses to reconstruct bone defects in our hospital from January 2014 to December 2020 were included in the study group Twelve matched patients who underwent sacroiliac joint tumor resection and reconstruction with bone cement and screws in the same time period were selected as the control group.

**Results:**

In the 3D-printing group, six cases were extensively excised, and six cases were marginally excised. All patients were followed up for 6–90 months, and the median follow-up time was 21 months. Among them, nine patients had disease-free survival, two survived with tumor recurrence, and one died due to tumor metastasis. The MSTS-93 score of the surviving patients was 24.1 ± 2.8. The operation time was 120.30 ± 14.50 min, and the intraoperative bleeding was 625.50 ± 30.00 ml. In the control group, seven cases were extensively excised, and five cases were marginally excised. All patients were followed up for 6–90 months, with a median follow-up time of 20 months. Among them, nine patients had disease-free survival, one survived with tumor recurrence, and two died due to tumor metastasis. The MSTS-93 score of the patients was 18.9 ± 2.6. The operation time was 165.25 ± 15.00 min, and the intraoperative bleeding was 635.45 ± 32.00 ml. There was no significant difference in survival status, intraoperative blood loss, or complications between the two groups (*P*>0.05). However, there were statistically significant differences in operative time and postoperative MSTS-93 scores between the two groups (*P*<0.05).

**Conclusions:**

After resection of the sacroiliac joint tumor, reconstruction using 3D printed prostheses was shorter and resulted in better movement function.

## Introduction

Indeed, iliosacral resection without reconstruction could serve as an effective treatment option for pelvic type I–IV tumors (1). But with the advances in adjuvant chemotherapy, imaging examination, and surgical techniques in recent decades, the safety and effectiveness of limb salvage surgery for pelvic tumors have been widely recognized (2). Currently, there are many methods for bone defect reconstruction after sacroiliac joint tumor resection; these can be divided into two categories: biological reconstruction and prosthesis reconstruction. There is no clear consensus on which method is the best (3). Biological reconstruction includes bone fusion, transposition, and inactivated replantation, and the advantages of this approach are that it can achieve permanent bone or scar healing, avoid prosthesis revision and other problems, and obtain satisfactory functional scores (4). However, there are some disadvantages of biological reconstruction, such as long-term immobilization, non-union, pseudoarticulation, and infection (5). Prostheses include bone cement and metal prostheses, and the application of prosthesis reconstruction can achieve early activity, good cosmetics, initial mechanical stability, and satisfactory function (6). However, potential complications include prosthesis loosening, infection, dislocation, and fracture (7, 8).

To overcome the disadvantages of protheses, improved surgical skills and new manufacturing processes or materials may be needed to optimize the mechanical properties and biocompatibility of prostheses. Further, 3D printing technology provides bone oncologists and engineers with more inspiration and freedom in the design of prostheses (1, 9–11). Theoretically, 3D printing technology can produce a prosthesis of any shape to achieve precise matching between the prosthesis and the osteotomy surface (10, 11). Simultaneously, a screw path in any direction can be reserved on the prosthesis to restore normal mechanical conduction (12). Porous surface structures that induce bone growth and fusion can also be produced (13, 14). Finally, biological reconstruction was performed on the basis of prosthesis reconstruction (2, 10, 11).

The purpose of this study was to retrospectively analyze and compare the data of 12 patients with sacroiliac joint tumors reconstructed by a 3D-printed prosthesis and 12 patients with sacroiliac joint tumors reconstructed by bone cement combined with screws, in order to explore the safety and effectiveness of the application of 3D-printed prostheses.

## Materials and Methods

### Inclusion and Exclusion Criteria

The inclusion criteria were as follows: in the 3D-printing group, from January 2014 to December 2020, patients with sacroiliac joint tumors who underwent tumor resection and 3D-printed prosthesis insertion to reconstruct bone defects were enrolled. In the control group, patients who underwent sacroiliac joint tumor resection and received bone cement combined with screws between January 2014 and December 2020 were enrolled in the study. Patients with the same or similar parameters as the 3D-printing group were screened out according to sex, age, height, weight, tumor size, region, and stage. The exclusion criteria for both groups were as follows: patients who did not receive surgical treatment or underwent reconstruction by other methods.

### Patients

The baseline data of the 24 patients in this study are listed in **Table 1**. Preoperative pelvic magnetic resonance imaging (MRI), chest computed tomography (CT), and single-photon emission computed tomography/computed tomography (SPECT/CT) were performed. All patients with osteosarcoma and Ewing’s sarcoma received standard preoperative neoadjuvant chemotherapy. 3D-printed prostheses were used in 12 cases in the 3D-printing group, and bone cement combined with screws was used in 12 cases in the control group.

**Table 1:** Baseline data of patients with sacroiliac joint tumor resection and reconstruction.

Item	3D-printing group (n = 12)	Control group (n = 12)	*P* value
**Sex** (n)			*P *> 0.05
Male	7	6	
Female	5	6	
**Age** ${(}^{\bar{x}}\pm s,\;years)$	42.5 ± 3.4	42.8 ± 2.8	*P *> 0.05
**BMI** ${(}^{\bar{x}}\pm \text{s},{\text{ kg/m}}^{2})$	22.6 ± 2.8	22.8 ± 2.5	*P *> 0.05
**Pathologic diagnosis (n)**			*P*> 0.05
Osteosarcoma	4	5	
Chondrosarcoma	2	1	
Ewing’s sarcoma	1	1	
Giant cell tumor of bone	1	1	
Other primary malignancies	1	2	
Metastasis	3	2	
**Gross tumor volume** ${(}^{\bar{x}}\pm \text{s},{\text{ cm}}^{3})$	327.2± 62.4	336.4± 61.9	*P *> 0.05

### 3D-Printed Prosthesis

**Figure 1:**
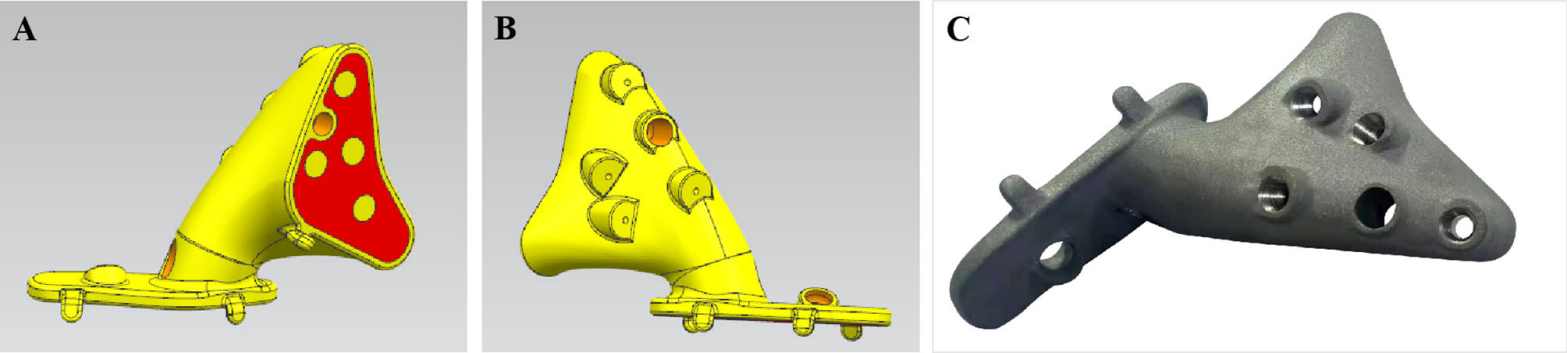
**(A–C)** 3D-printed designs and drawings of the prostheses.

The osteotomy guide plate and prosthesis were customized for each patient and produced (Thytec, Co, Ltd, Shanghai, China) according to the preoperative design, which took approximately 7 to 14 days. As previously reported (9, 10), an osteotomy guide plate for accurate tumor resection and a personalized prosthesis for perfect fit of the bone defect were then designed (**Figure 1**).

### Tumor Resection and Reconstruction

**Figure 2:**
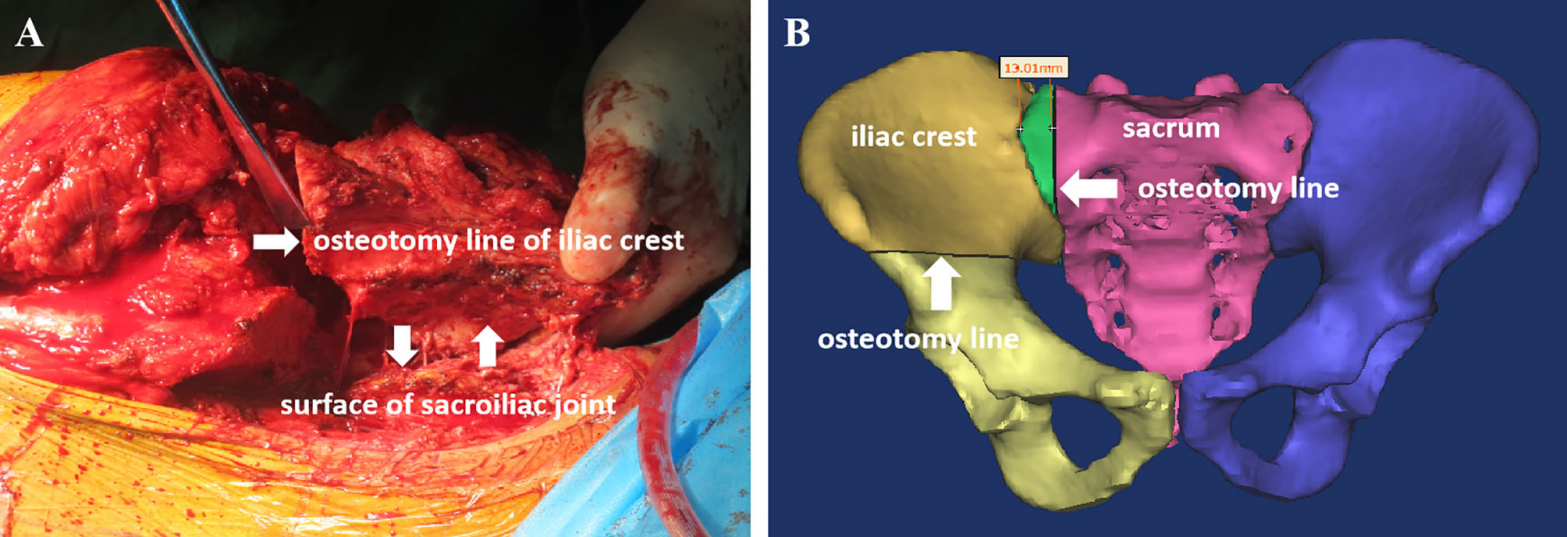
Diagram of resection of iliac bone and sacroiliac joint. **(A)** The tumor is separated and resected by separating the sacroiliac joint. **(B)** For tumors involving the sacrum, part of the sacrum is removed, while the sacral nerves are preserved.

**Figure 3:**
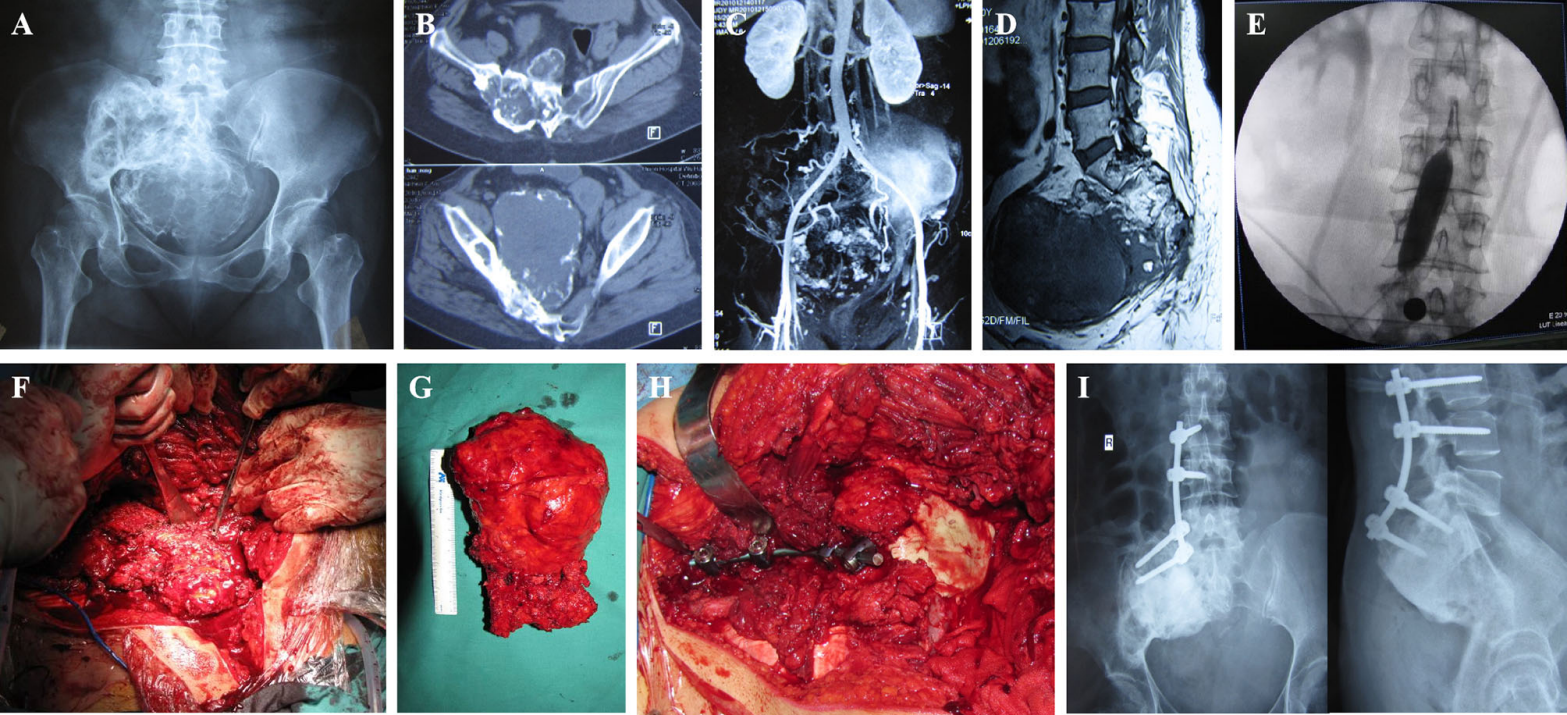
A 50-year-old female patient with a giant cell tumor of bone. **(A)** Preoperative X-ray shows a right sacroiliac joint tumor. **(B)** Preoperative computed tomography (CT) shows a right sacroiliac joint tumor. **(C)** Angiography showing abundant blood supply in the tumor. **(D)** Magnetic resonance imaging (MRI) showing mixed signals in the tumor. **(E)** Abdominal aorta balloon block is used to control surgical bleeding. **(F)** En bloc resection of the tumor. **(G)** General view of the specimen. **(H)** Bone cement combined with lumbar iliac screw fixation. **(I)** Twenty-seven months postoperative X-ray shows no loosening or fracture of screws and connecting rods.

**Figure 4:**
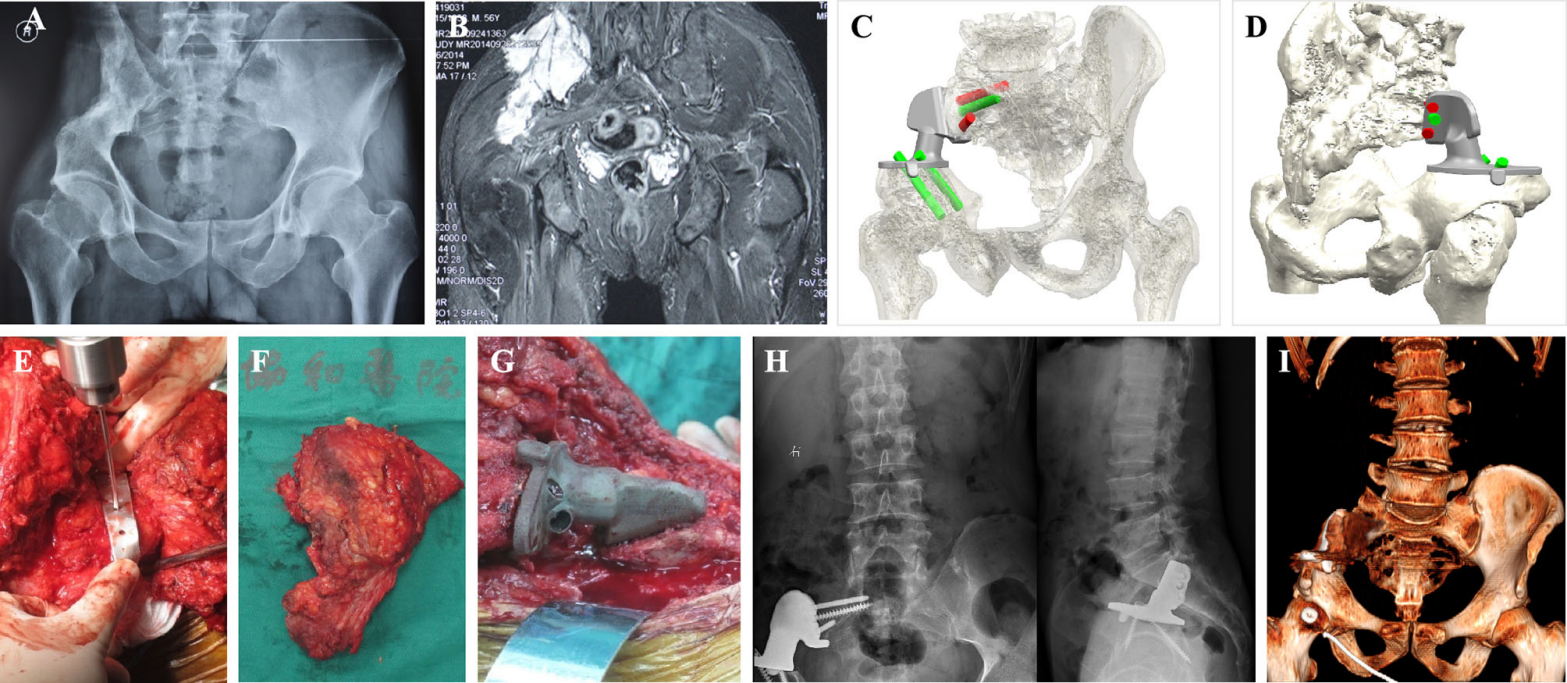
A 56-year-old male patient with recurrent osteosarcoma. **(A)** Preoperative X-ray shows a right sacroiliac joint tumor. **(B)** Preoperative magnetic resonance imaging (MRI) shows a right sacroiliac joint tumor. **(C, D)** Design and simulated installation of 3D-printed prosthesis. **(E)** Installation of 3D-printed osteotomy guide plate. **(F)** General view of the specimen. **(G)** Installation of 3D-printed prosthesis. **(H)** Twenty-five months postoperative X-ray shows the prosthesis was fitted precisely to the residual bone. **(I)** Twenty-five months postoperative computed tomography (CT) showed significant bone in-growth at the bone-prosthesis interface.

**Figure 5:**
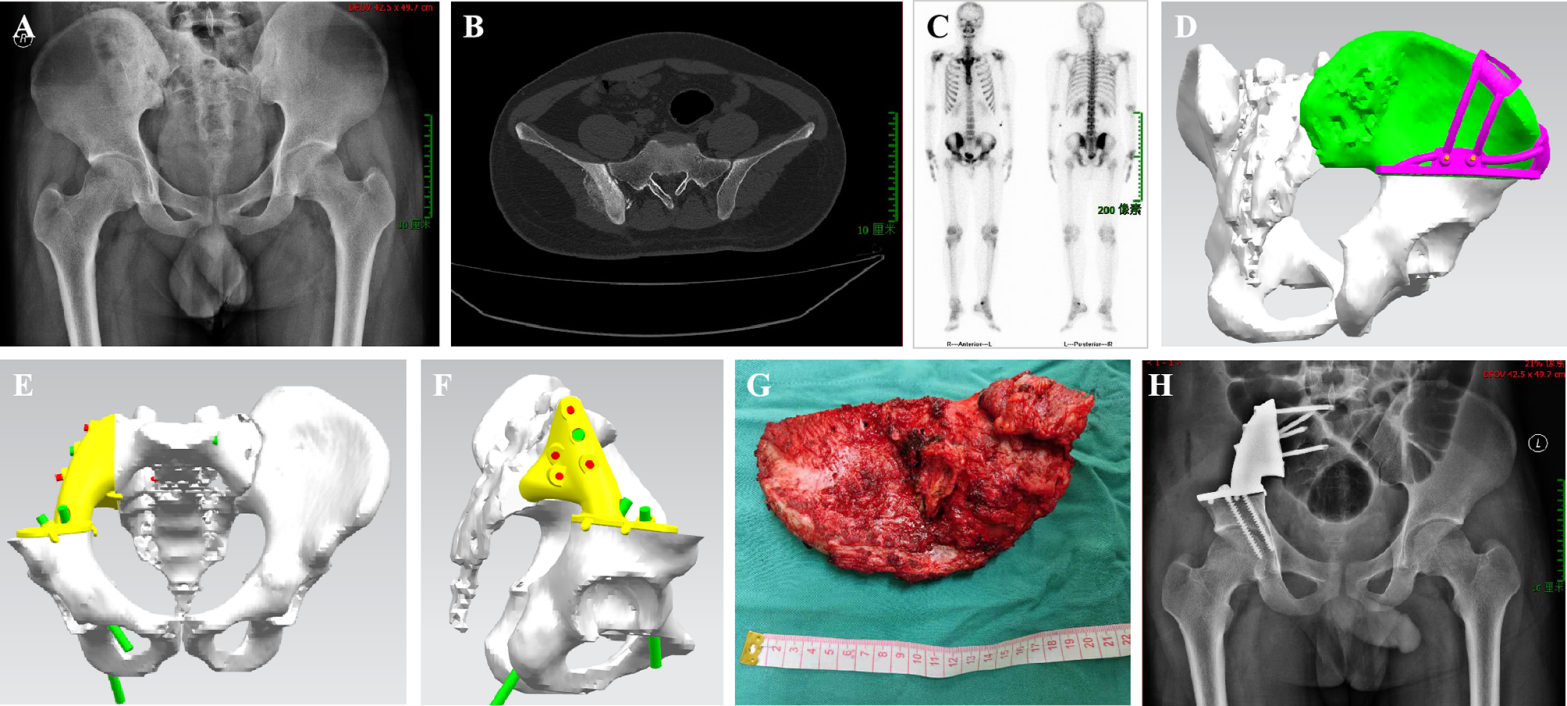
A 22-year-old male patient with osteosarcoma. **(A)** Preoperative X-ray shows a right sacroiliac joint tumor. **(B)** Preoperative computed tomography (CT) shows a right sacroiliac joint tumor. **(C)** Single-photon emission computed tomography/computed tomography (SPECT/CT) showing active metabolism in the tumor. **(D–F)** A 3D-printed model is used for osteotomy guide-assisted resection of the tumor and simulates the installation of the prosthesis. **(G)** A gross specimen of resected tumor. **(H)** Twenty-seven months postoperative X-ray shows stable internal fixation.

The iliac crest was removed based on the preoperative plan. The sacroiliac joint was gradually expanded while the sacroiliac joint was cut open, and the sacroiliac joint was removed after complete separation (**Figure 2A**). For tumors involving the sacrum, part of the sacrum was removed, while the sacral nerves were preserved (**Figure 2B**). Tumor resection has been described in previous reports (1, 9, 10). In the control group, after resection of the tumor, pedicle screws and iliac screws were placed in the L4 and L5 vertebral pedicles and the iliac wing, and then the titanium rod was placed. After the reconstruction of the nail rod system was completed, the bone defect was filled with bone cement (**Figure 3**). In the 3D-printing group, after resection of the tumor, the prosthesis was placed on the residual iliac crest with the base attached to the outer plate of the iliac crest and the osteotomy surface. Screws were placed in the S1 and/or S2 vertebrae and the iliac crest *via* the screw passage to reinforce the fixation of the prosthesis (**Figures 4**, **5**).

### Postoperative Treatment

After the operation, anti-infection treatment was applied, and an inflatable leg pump was used to prevent venous thrombosis of the lower limbs. The drainage tube was removed when the daily drainage volume was less than 50 ml. After the drainage tube was removed, the patient was encouraged to walk and undergo rehabilitative training.

### Follow-Up Plan and Efficacy Evaluation

Patients were required to have outpatient follow-up visits 3, 6, and 12 months after the surgery for pelvic X-ray re-examination. After 1 year, the pelvic plain radiograph was once every 6 months, and after 3 years, the X-ray was performed once a year. The Musculoskeletal Tumor Society (MSTS) functional scoring system was used to assess lower-limb function (15). Tumor recurrence, loosening of internal fixation, and fracture were evaluated according to the pelvic plain film.

### Statistical Analysis

The patient’s pain level and limb function were analyzed using SPSS 20.0 (IBM, USA). An χ^2^ test was used to compare the classification variables between the groups. An independent sample *t*-test was used for intergroup comparisons of continuous variables, and a paired *t-test* was used to compare the MSTS scores. Statistical significance was set at *P*<0.05.

## Results

### Comparison of Baseline Data

There were no statistically significant differences in age, sex, body mass index (BMI), pathological type, and tumor volume between the 3D-printing group and the control group (*P*>0.05) (**Table 1**).

### Surgical Results

In the 3D-printing group, six cases were extensively excised and six cases were marginally excised. The operation time was 120.30 ± 14.50 min, and the intraoperative bleeding was 625.50 ± 30.00 ml. In the control group, seven cases were widely excised, and five cases were marginally excised. The operation time was 165.25 ± 15.00 min, and the intraoperative bleeding was 635.45 ± 32.00 ml. There were no deaths due to perioperative complications in either group. There was no significant difference in intraoperative blood loss (*P*>0.05), but there was a statistically significant difference in operative time between the two groups (*P*<0.05) (**Table 2**).

**Table 2:** Surgical data and follow-up results of patients with sacroiliac joint tumor resection and reconstruction.

Item	3D-printing group (n = 12)	Control group (n = 12)	*P* value
**En bloc resection **(n)	12	12	*P *> 0.05
**Surgery boundary** (n)			*P *> 0.05
Wide excision	6	7	
Margin excision	6	5	
**Operating time** ${(}^{\bar{x}}\pm \text{s},\text{ min})$	120.30 ± 14.50	165.25 ± 15.00	*P *< 0.05
**intraoperative bleeding** ${(}^{\bar{x}}\pm \text{s},\text{ ml})$	625.50 ± 30.00	635.45 ± 32.00	*P *> 0.05
**Bleeding control method** (n)			*P *> 0.05
Abdominal aorta balloon block	5	4	
Selective arterial embolization	7	8	
**Median follow-up** (months)	21	20	*P *> 0.05
**Survival status** (n)			*P *> 0.05
Survival with disease free	9	9	
Survival with tumor	2	1	
Died	1	2	
**Complication** (n)	1	1	*P *> 0.05
**MSTS-93 score** ${(}^{\bar{x}}\pm \text{s},\text{ score})$	24.1 ± 2.8	18.9 ± 2.6	*P *< 0.05

### Oncology Results

In the 3D-printing group, all patients were followed up for 6 to 90 months, with a median follow-up time of 21 months. Among them, nine patients had disease-free survival, two survived with tumor recurrence, and one died due to tumor metastasis. The patient died from osteosarcoma, and the time of death was 13 months after surgery. In the control group, all patients were followed up for 6 to 90 months, with a median follow-up time of 20 months. Among them, nine patients had disease-free survival, one survived with tumor recurrence, and two died due to tumor metastasis. One of the deaths was osteosarcoma, and the other was a lung metastasis of osteosarcoma. For these two patients, the mean survival post-surgery was 14 months (10–18 months) (**Table 2**).

### Postoperative Functional Status

Functional ratings were assessed using the MSTS-93 scale. In the 3D-printing group, the MSTS-93 score of the surviving patients ranged from 18 to 28, and the mean score was 24.1 ± 2.8. In the control group, the MSTS-93 score of the patients ranged from 12 to 24, and the average score was 18.9 ± 2.6. There was a statistically significant difference in the postoperative MSTS-93 scores between the two groups (*P*<0.05) (**Table 2**).

### Postoperative Complications

Delayed wound healing occurred in one case in the 3D-printing group, and one patient had screw rupture in the control group The delayed healing wound was debrided and healed after 2 weeks. There was no significant difference in complications between the two groups (*P*>0.05).

## Discussion

The sacroiliac joint is a fretting-joint composed of the sacral auricular surfaces and the ilium. The motion range of the joint decreases with age and generally disappears at age 40–50 (16). The integrity of the sacroiliac joint composite structure is the basis for the integrity of the pelvic ring. When the stability of the sacroiliac complex is damaged, the sacroiliac joint is prone to rotation or vertical shear instability under pelvic load conditions, which is likely to lead to sacroiliac pain (17). Synovial metabolism is fast on the sacral surface of the sacroiliac joint, and the blood supply is rich; therefore tumors, tuberculosis, and other lesions are mostly located on the side of the sacrum (18). Whether reconstruction is beneficial after iliosacral tumor resection remains controversial (19). Jin et al. suggest that because of the high rates of complications and recurrence, some patients cannot benefit from reconstruction. They found that iliosacral resection without reconstruction removed more than one-third of the I-A distance, leading to a limb-length discrepancy and degraded acetabular coverage without altered functional outcome. Indeed, iliosacral resection without reconstruction could serve as an effective treatment option for pelvic type I–IV tumors (1). Gordon et al. reported on 16 patients who underwent resection of the iliosacral joint; all 4 patients who underwent reconstruction required walking aids, whereas among the 12 patients who did not undergo reconstruction, 9 were able to walk without aids (20).

After the tumor was excised, the sacroiliac joint was fixed with the pedicle system *via* the lumbar iliac. This operation was based on the improved Galveston technique. The L4 and L5 pedicles were fixed with pedicle screws, and the iliac bone was fixed with two iliac bone screws to restore the stability of the lumbar sacroiliac joint and achieve stability of the posterior pelvic ring. Biomechanical studies have shown that this type of reconstruction has very strong stability. The pedicle is one of the hardest parts of the spine, and the pedicle screw has good resistance to pull-out force and shear force, and can achieve three-dimensional fixation (21). Zhou et al. retrospectively reviewed 16 patients who underwent pelvic prosthesis reconstruction using the pedicle screw-rod system after pelvic resection for primary sacroiliac joint tumors, and found that it is characterized by easy manipulation, few complications, and stable fixation (22).

Some studies have pointed out that although unilateral fixation can provide sufficient fixation in the early postoperative period, the movement of the sacroiliac joint increases the stress on the tail of the titanium rod and the iliac nail connecting the L5 to the iliac crest, shortening the anti-fatigue life of the internal fixation device and increasing the risk of nail fracture (23, 24). Selecting the appropriate lumbar segment for fixation is critical for maintaining the effectiveness of internal fixation. In this study, we chose L4 and L5 pedicle screw placement instead of extending the fixation segment upward because this is closest to the normal biomechanical state and so can effectively maintain the stability of internal fixation. Wang et al. examined the outcomes of patients who underwent extensive resection of periacetabular tumors involving the sacroiliac joint and joint reconstruction with a hemipelvic endoprosthesis. However, they found that adding an extra screw for fixation to the S1 vertebra was not associated with any improvement in clinical outcomes during a short-term follow-up period (6).

In this study, the 3D-printed prostheses had a good safety profile, with simple intraoperative installation and fewer postoperative complications. Compared with the control group, the use of a 3D-printed prosthesis reduced the operative time. The easy installation of 3D-printed prostheses is due to their highly anatomical shape design and the inclusion of the screw path in a fixed direction, so that the location of the prosthesis can be found quickly and the screws can be placed accurately after osteotomy. In this study, 3D-printed osteotomy guide plates were used to define tumor resection, and they achieved excellent matching between the prosthesis and residual bone fragments (1, 10–13). Moreover, the application of 3D-printed osteotomy guide plates can reduce the operation time.

The 3D-printed prosthesis is more suitable for the curved shape of the iliac outer plate, and the screws can be accurately placed into the S1 and/or S2 vertebrae. Additionally, the use of long cancellous bone screws and short cortical screws to fix the prosthesis can achieve more stable fixation and natural mechanical conduction (10, 11). In this study, the 3D-printed prosthesis was designed as 1.5 mm thick porous titanium interface. The pore size ranged from 450 to 550 μm, and the porosity was approximately 60%. This prosthesis design is beneficial for bone growth and bone fusion. The MSTS-93 score of the surviving patients treated with a 3D-printed prosthesis was 24.1 ± 2.8, and there was a statistically significant difference in postoperative MSTS-93 scores between the 3D-printing and control group. One patient had a screw rupture in the control group; however, the patient did not develop severe lower limb dysfunction and therefore did not undergo any revision surgery. This study showed that 3D-printed prostheses can not only allow for early functional exercise and reduced complications but can also achieve “mechanical biological” reconstruction. While 3D-printed prostheses offer many advantages, older technologies such as bone cement combined with screws are still acceptable when 3D-printing technology is not available. There was no significant difference in survival status, intraoperative blood loss, and complications between the two groups in our study (*P*>0.05).

This study has some limitations. First, this was a retrospective case-control study, which inevitably suffered selection bias. Second, the overall postoperative follow-up time was short, which may have underestimated the incidence of mechanical complications such as aseptic loosening and screw fracture. Third, this was a small-sample study in a single center, and a large study in multiple centers is needed. Finally, the effect of the 3D-printed prosthesis on bone growth needs to be confirmed by a longer follow-up period.

## Conclusion

The results of this retrospective case-control study of 3D-printed sacroiliac joint prostheses suggest that the use of 3D-printed prostheses to reconstruct bone defects after sacroiliac joint tumor resection can achieve good safety and functional status. However, the long-term prognosis requires further follow-up.

## Data Availability Statement

The original contributions presented in the study are included in the article/supplementary material. Further inquiries can be directed to the corresponding authors.

## Ethics Statement

This study was approved by the Ethics Committee of Union Hospital, Tongji Medical College, Huazhong University of Science and Technology, and all patients signed informed consents. The patients/participants provided their written informed consent to participate in this study. Written informed consent was obtained from the individual(s) for the publication of any potentially identifiable images or data included in this article.

## Author Contributions

ZC and ZS designed the study and supervised the report. DS, XH, JZ, BW, and QW performed the research and analyzed the data. FP and JL drafted the manuscript. All authors contributed to the article and approved the submitted version.

## Funding

This study was supported by the National Natural Science Foundation of China (grant no. 81904231) and the China Postdoctoral Science Foundation (grant no. 2020M672369).

## Conflict of Interest

The authors declare that the research was conducted in the absence of any commercial or financial relationships that could be construed as a potential conflict of interest.

The handling editor declared a shared parent affiliation with the authors at time of review.

## Publisher’s Note

All claims expressed in this article are solely those of the authors and do not necessarily represent those of their affiliated organizations, or those of the publisher, the editors and the reviewers. Any product that may be evaluated in this article, or claim that may be made by its manufacturer, is not guaranteed or endorsed by the publisher.
